# Editorial

**Published:** 1870-06

**Authors:** 


### Editorial.

Proceedings of Medical Societies.—We had intended to publish in the present number an abstract of the proceedings of the American Medical Association, and of the Illinois State Medical Society. But the full record of proceedings of the Convention of Delegates, from Medical Colleges, recently held in Washington, occupies so much space that we shall be compelled to defer that of the national and state organizations until the next number. In the meantime, we will simply state that the meeting of our State Society in Dixon, on the 17th and 18th of May, was one of the most pleasant and profitable that its members have ever enjoyed. Though many of the Committees were delinquent in regard to reports, yet all the time of the meetings was occupied in the hearing and discussion of papers and questions of importance.

The Committee of Arrangements, in conjunction with the people of Dixon, received the members of the Society with cordial hospitality, and on Wednesday afternoon, gave them a delightful entertainment in the form of a Picnic; on the grounds of Mr. Cherters, located in the bend of Rock River, elevated abruptly one hundred feet or more, and affording one of the most beautiful places that we have visited. The Mayor, the good Ladies of Dixon, the proprietor of the Nachusa House, and the Committee of Arrangements, with its venerable Chairman, will long be remembered with pleasure by those who participated in the meeting at Dixon.

In regard to the recent meeting of the American Medical Association, at Washington, we have space to say only a few words. In numbers, it was equal to any previous meeting. In the scientific part of its work (which is all done in the several sections), it will compare favorably with any of the previous meetings, as the volume of Transactions, when published, will show. The general meetings, which occupy only a part of each day, and which are devoted to the hearing of reports from the Officers and Standing Committees, and the transaction of miscellaneous business were well attended, and resulted in the accomplishment of the usual business in proper order. The only cause of serious annoyance during the recent meeting was the local controversy in the District of Columbia, arising from the attempt, on the part of a few members of the profession, to organize a new Medical Society, coincidently with an effort in Congress to abrogate the Charter of the old Society of the District. Some of the parties to this controversy succeeded in keeping up just enough agitation of it, in the general sessions of the Association, to annoy and disgust many who felt little interest in the local quarrel.

But even this did not create more disturbance than had been induced by other causes, during several previous meetings of the Association. The daily reports in the *Chronicle* and other

Washington newspapers, were simply grossly exaggerated partizan misrepresentations, full of errors both of omission and commission, and we regret to see them copied into the weekly medical journals of Philadelphia, New York, and Boston.

---

American Medical Association, Record of its Proceedings.—We are informed that Dr. W. B. Atkinson, permanent Secretary of the Association, proposes to publish an edition of the proceedings of the recent meeting of the Association, in pamphlet form, for twenty-five cents per copy. We hope all members of the profession who desire a *correct* account of the doings of the Association, will send him their names without delay. His address is, Wm. B. Atkinson, M.D., S.-W. Corner of Broad and Pine Streets, Philadelphia.

---

Allen County Medical Society.—We had the pleasure of attending the annual meeting of this Society yesterday, May 31st, at Fort Wayne, Indiana. The number in attendance was large, evincing an active interest in professional advancement highly commendable. Several highly interesting cases of diseases and accidents were presented to the Society, followed by a discussion on scarlet fever, in which most of the members participated. After the public address in the evening, an elegant entertainment was given at the McKenzie House, which was highly enjoyed by all. We ought to have many more such societies.

---

Professional Quarrels.—"In New York, Boston, Chicago, and some other cities, members of the medical profession are fully engaged in professional and personal quarrels."—*Buffalo Medical and Surgical Journal.*

We clip the above from an editorial in the April number of the *Buffalo Medical and Surgical Journal*, for the purpose of asking our confiéré, why he perpetrated such a slander upon the profession of this goodly city. We know of no quarrels in the profession here, and have known none for the last ten years. On the contrary, we think it would be difficult to find any city in which there is less professional bickering or controversy than here. We think our neighbor in Buffalo must be mistaken in his geography.

---

### Mortality for the Month of April, 1870.

| | |
|---|---|
| Accident, burned by petrolium | 1 |
| " drowned | 1 |
| " fall | 2 |
| " foreign body in trochea | 1 |
| " overdose soothing syrup | 1 |
| " wound, gun-shot, of chest | 1 |
| " run over by wag. | 1 |
| " scalded | 1 |
| " railroad | 1 |
| Abscess, pelvic | 1 |
| Anæmia puerperal | 1 |
| Apoplexy | 2 |
| " abscess of brain | 1 |
| Ascitis | 1 |
| " cirrhosis of liver | 1 |
| Asphyxia | 1 |
| Asthma and myelitis | 1 |
| Atelectasis pulmonum | 1 |
| Aneurism, abdominal | 1 |
| Bowels, cancer | 2 |
| Brain, congestion | 9 |
| " inflammation | 4 |
| Bronchitis | 14 |
| " & complications | 2 |
| " chronic | 1 |
| " capillary | 3 |
| Carditis | 1 |
| Cancer of back | 1 |
| Childbirth | 2 |
| Consumption | 57 |
| Convulsions | 50 |
| Croup | 7 |
| " diphtheretic | 1 |
| " membranous | 3 |
| Cynanche tonsillaris | 1 |
| Cyst, hepatic | 1 |
| Debility, general | 3 |
| Delirium tremens | 2 |
| Diarrhœa | 3 |
| " chronic | 1 |
| Diphtheria | 9 |
| Dropsy, general | 4 |
| " of abdomen | 1 |
| " " chest | 2 |
| Drowned | 4 |
| Dysentery | 3 |
| " acute | 1 |
| " chronic | 1 |
| Embolism, fr'm wound of foot | 1 |
| Endocarditis | 1 |
| Enteritis | 7 |
| Erysipelas | 5 |
| Fever, congestive | 2 |
| " puerperal | 5 |
| " remittent | 2 |
| " scarlet | 27 |
| " " complications | 3 |
| " " malignant | 7 |
| " typhoid | 8 |
| Gastritis and entero-colitis | 1 |
| Hemorrhagia, general | 1 |
| Hemorrhage, cerebral | 1 |
| Heart, dropsy, result of angina pectoris | 1 |
| " valvular disease | 4 |
| Hemiplegia | 1 |
| Hepatitis, chronic | 2 |
| Hernia, incarcerated | 1 |
| Hydrocephalus | 12 |
| " acute | 1 |
| " chronic | 1 |
| Hydro-pericarditis | 1 |
| Inanition | 16 |
| Intemperance | 1 |
| Kidneys, disease of | 1 |
| " Bright's " | 5 |
| Laryngitis | 1 |
| Liver, cirrhosis | 1 |
| " cancer, & dropsy of abdomen | 1 |
| Lungs, congestion | 4 |
| " " and heart, fatty degeneration | 1 |
| " emphysema | 1 |
| " hemorrhage | 2 |
| Measles | 7 |
| " & complications | 7 |
| Metro-peritonitis | 1 |
| Meningitis | 12 |
| Meningitis, cerebro-spinal | 6 |
| Metritis | 1 |
| " puerperal | 2 |
| Necrosis of sacrum, result of fall | 1 |
| Nephritis, chronic | 1 |
| Neck, tumor | 1 |
| Old age | 5 |
| Paralysis | 2 |
| Paraplegia | 1 |
| " result of injury to spinal cord | 1 |
| Peritoneal inflammation, acute, from gall stone | 1 |
| Peritonitis | 3 |
| " following operation for stone in bladder | 1 |
| " chronic & dropsy | 1 |
| Pleurisy | 2 |
| Pneumonia | 49 |
| " broncho | 1 |
| " & complications | 2 |
| " typhoid | 4 |
| Pyæmia | 1 |
| Scrofula | 1 |
| " of head | 1 |
| " enlargement of bronchi'l glands | 1 |
| Small-Pox | 3 |
| Spina bifida | 1 |
| Spine, caries | 1 |
| Stomach, cancer | 2 |
| Suicide, laudanum | 1 |
| Syphilis, congenital | 1 |
| " hereditary | 1 |
| Tabes mesenterica | 10 |
| Teething | 3 |
| " & complications | 2 |
| Thrombosis, puerperal | 1 |
| Uterus, cancer of | 2 |
| Unknown | 1 |
| Whooping-cough | 5 |
| " & complications | 1 |
| Total | 494 |

### COMPARISON.

| | | | |
|---|---|---|---|
| Deaths in April 1870, 494 | Deaths in April 1869, 381 | Increase, | 113 |
| Deaths in March, 1870, | 537 | Decrease, | 43 |

AGES.

| | | | | | |
|---|---|---|---|---|---|
| Under 1 | 154 | 10 to 20 | 14 | 60 to 70 | 13 |
| 1 to 2 | 72 | 20 to 30 | 4 | 70 to 80 | 7 |
| 2 to 3 | 25 | 30 to 40 | 42 | 80 to 90 | 5 |
| 3 to 4 | 18 | 40 to 50 | 29 | | |
| 4 to 5 | 15 | 50 to 60 | 17 | Total, | 494 |
| 5 to 10 | 34 | | | | |

| | | | | | |
|---|---|---|---|---|---|
| Males, | 280 | Females, | 214 | Total, | 494 |
| Single, | 375 | Married | 119 | Total, | 494 |
| White, | 481 | Colored, | 13 | Total, | 494 |

NATIVITY.

| | | | | | |
|---|---|---|---|---|---|
| Bohemia | 5 | Germany | 66 | Scotland, | 2 |
| Canada | 6 | Holland | 5 | Sweden | 8 |
| Chicago, Native | 78 | Ireland | 45 | Switzerland | 1 |
| Chicago, Foreign | 190 | Norway | 4 | Unknown | 3 |
| U. S., other parts | 64 | New Brunswick | 1 | | |
| Denmark | 1 | Poland | 2 | Total, | 494 |
| England | 13 | | | | |

MORTALITY BY WARDS FOR THE MONTH.

| Wards. | Mortality. | | Mortality. |
|---|---|---|---|
| 1 | 10 | Unknown Ward | 1 |
| 2 | 17 | Accidents | 10 |
| 3 | 18 | County Hospital | 11 |
| 4 | 13 | Convent of Good Shepards | 1 |
| 5 | 10 | Home for Friendless | 3 |
| 6 | 25 | Hospital Alexian Brothers | 2 |
| 7 | 25 | Half Orphan Asylum | 1 |
| 8 | 47 | Immigrants | 1 |
| 9 | 42 | Jewish Hospital | 2 |
| 10 | 13 | Luke Hospital | 2 |
| 11 | 23 | Lutheran Hospital | 1 |
| 12 | 17 | Lake Basin | 1 |
| 13 | 7 | Mercy Hospital, | 4 |
| 14 | 15 | Protestant Orphan Asylum | 4 |
| 15 | 36 | St. Luke Hospital | 2 |
| 16 | 28 | St. Joseph Orphan Asylum, | 11 |
| 17 | 26 | Suicide | 1 |
| 18 | 34 | | |
| 19 | 16 | Total, | 494 |
| 20 | 14 | | |

---

DR. DIEULAFOY'S "ASPIRATEUR SOUSCUTANE."—Under this name an instrument has been suggested, by means of which effusions into synovial or serous membranes, collections of pus or blood, and even hydatid sacs may be safely evacuated. It consists of an instrument resembling a subcutaneous injection syringe, with a terminal and a lateral tube fitted with stop-cocks, to which a *capillary* trocar can be fitted, so that after withdrawal of the morbid liquid an injection may be practiced, without removing either the trocar or the pump.—*Medical Times and Gazette, Nov. 20th, 1869.*

Quackery and the Press.— * * * "The most powerful auxillary of quackery is, undoubtedly the press; in fact the nostrum-vendors depend so entirely upon it for support in the way of advertising themselves, that without it they could do nothing. The press, in its turn, as a matter of emolument, opens its columns freely and unreservedly to any who choose to pay the cash price for such a privilege of an insertion. This practice is, as is well known, not confined to any particular class of periodicals, for even the most respectable of them eagerly bite at the bait. We doubt whether there can be found to-day, over this country of ours, more than half-a-dozen journals, religious or political, that dare refuse admission into their advertising columns of any quack nostrum that may be offered. On other points they may be orthodox enough; very many will not advertise licentious books, improper places of amusement, and not a few religious papers will go so far as to refuse the announcement of new works of fiction; but at the same time column after column of testimonials of cure by this and that remedy will appear with every issue."

We copy the above from the *Medical Record* (New York), both to endorse most fully all that it says in relation to the evils of advertising "quackery," and to point a moral to the tale by asking the *Record* to pluck the beam out of its own eye. It should call a blush to the entire medical press of this country that *money* is able to purchase the use of their columns for advertising some of the worst quackery known to medicine. The newspaper is *not* a medical journal; it does not assume to educate the people in this department; but the medical journal does assume to educate and inform the profession, which, in turn, is to instruct the people. We are gravely told, in the article quoted from above, that "the safest way to give medical facts to the community is to quote them from some medical journal of standing." Which are more worthy of confidence, the opinions of lawyers and clergymen of "troches" and "soothing syrup," or the published certificates of medical men attesting the wonderful virtues of some secret compound? How about "sweet quinine," "cincho-quinine," "svapnia," "cod-liver extract," tolu anodyne," "elixir of opium," together with sundry other "elixirs," and secret proprietary articles, all bearing heavy scientific names, in which their science principally consists, save only in the art of inducing medical journals to recommend them. Alas! is "the temptation of money too great to be resisted," or shall we see a reform in this matter? A sin may be measured by the light sinned against; by this guage, which is the more guilty, the newspaper, that knows nothing of medicine, or the medical journal, that professes to be a guide in all such matters? We hold the latter to be; and while it may not advertise female pills, it yet offers its endorsement to a class of medicines equally unworthy of confidence, and which it advertises (with *cross-line devices* borrowed from the quack advertisers in the newspapers) for precisely the same reason—to make money. The effect is also similar, as many poorly informed physicians use these nostrums, believing they have found at last the elixir vitæ. While we uphold the *Medical Record* in its position, we hope it will aim its next broadside at the "medical press," and, in its usually skilful manner, strike another blow at quackery.—*Pharmacist.*

---

Enlarged Tonsils Removed without Cutting.—Dr. A. Ruppaner reports (*Phil. Medi. & Surg. Reporter*) 123 cases of enlarged tonsils removed without cutting. In reference to the many objections to the operation of excision, made by parents or friends and often fostered by physicians, he says, "Dr. James Yearsley, of London, has operated in more that *three thousand cases* without asingle accident, or a single unfavorable result from excision. The testimony of other distinguished surgeons is no less positive. In my own practice I have operated on several hundred patients without a single accident, nor was a solitary case followed by unfavorable or injurious consequences." There are cases, however, in which excision is impracticable, and in which nitrate of silver and iodine have been recommended. Dr. Ruppaner is of opinion that these are useless. Two much more active and satisfactory remedies proposed by Dr. Fournie, of Paris, are *Vienna paste* and *bichromate of potassa*, the former of which is preferred. A better preparation still is the *London paste*, proposed and named by Dr. M. Mackenzie, and consisting of equal parts of *caustic soda and lime*, moistened with a little absolute alcohol. The caustic lime and soda, finely pulverized and intimately mixed, is kept in a well-stoppered bottle. "When an application is to be made to the tonsils, a little of the powder is put into a small, porcelain cup, a few drops of absolute alcohol, which is kept near at hand, are added, the two are carefully mixed with a glass rod, when the paste is ready for use. Care must however be taken that it be of the proper consistency. If too thin, it is apt to find its way to parts which ought not to be touched; if too thick or lumpy, the paste will not readily stick, and little pieces might be swallowed. To apply the paste, a glass rod of sufficient length ought to be used. The end of it, which must be smooth and slightly funnel-shaped, is dipped into the paste, and a greater or lesser portion of the surface touched, as occasion may require." It is better that the patient should be in the position for laryngoscopy, when the paste is applied. Its action is very rapid, and occasions but little pain. The mucous membrane almost instantly assumes a deep flesh color, and presently a dark, blackish patch is seen, streaked with blood. The following day the tonsil is covered with a whitish yellow eschar. The operation is to be repeated in two or three days. The minimum number of applications in the 123 cases was 6, the maximum 14, and the time of treatment extended from three weeks to two and a half months.—*Pacific Medical and Surgical Journal.*

---

Effects of Tobacco-Smoking on Children.—It seems that the habit of smoking has taken possession of the boys in France to such an extent as to elicit serious inquiry as to its results. An able writer describes his experience on the subject, and comes to the following conclusions:

1. The pernicious effects on boys are incontestable.
2. They consist of pallor, chloro-anemia, palpitations of the heart, diminution of the normal number of red globules, and impaired digestion.
3. The ordinary treatment for anemia, etc., is ineffectual so long as the habit of smoking is persisted in.
4. Boys who are addicted to smoking exhibit a want of intelligence, and have a liking, more or less decided, for strong drink.
5. Those who abandon the practice before any serious organic lesions are produced, recover their health perfectly.

This subject concerns hundreds and thousands of boys in California. We may see daily, in the streets of San Francisco, scores of little human apes, scarcely old enough to dispense with diapers, puffing cigaritos and long nines, with all the airs of their exemplary parents. Beardless youths strut everywhere in thoroughfares and public places, sucking their sweet Havanas and meerschaums, and illustrating the benefits by a disgusting display of sunken eyes, lank cheeks, and bestial mouths—degenerating into animals, instead of growing up to manhood.—*Pacific Medical and Surgical Journal.*

---

COLLODION IN ENURESIS.—Sir D. J. Corrigan, Ireland, calls attention to a mechanical treatment of this trouble, in which, while the prepuce slightly curved up is held with the left hand, the surgeon smears over the little cup thus formed by the extremity of the prepuce, with collodion, by means of a small camel's-hair pencil, or blunt end of a penholder. Almost as fast as applied, the collodion solidifies. In contracting, it draws closely together the edges of the prepuce, and thus the exit for the escaping urine is closed. A boy of eleven years of age has, after one lesson, been able to use the collodion. A fortnight's use is sometimes sufficient for the cure. A relapse is easily dealt with. When the child or youth desires to pass water, the little wedge or cap of collodion is easily removed with the finger nail. When "I first used this collodion application, my expectation was that the bladder would act so forcibly against it as to cause sudden pain, and oblige the patient to jump at once out of bed and quickly remove the collodion, and that he should then repeat the application before returning to sleep. I was agreeably disappointed. There was no pain; no awaking; but on rising in the morning, the prepuce was found slightly distended with urine, and the collodion was removed without difficulty." It is most easy of application, occupies scarcely a minute, and can be carried out at school or elsewhere in perfect privacy.—*Dublin Quarterly Jour. of Med. Science.—Am. Practitioner.*

---

EXCISION OF KIDNEY.—Dr. Simon, of Heidelberg, has performed a surgical operation of considerable interest. In a woman upon whom he had performed ovariotomy there remained a flow of liquid issuing from a distinct situation in the abdominal cicatrix. All remedial proceedings, and even autoplasty, were successfully tried to no purpose. The character of the fluid was then investigated, and was found to be urine, proceeding from a lesion of the ureter which had occurred during the operation. Dr. Simon then undertook a series of experiments on animals with the object of determining whether a kidney might be removed without any evil consequences to the economy. The end of these researches was the decision to extirpate the kidney, and the operation has been performed with the most satisfactory results.—*Lancet, Feb. 3, 1870.*

---

CYNANCHE TONSILLARIS.—Dr. F. P. Atkinson, London, treats this troublesome affection in the following way: Bicarbonate of potash, one scruple; powdered guaiacum, ten grains, or tincture of guaiacum, half a drachm; mucilage, as required; water to the ounce; with fifteen grains of citric acid three times a day, in a state of effervescence. A gargle of twenty minims of tincture of iodine to the ounce of water (to be used by being held in the mouth and the head shaken from side to side). Three or four glasses of port-wine daily, and plenty of beef-tea. If the weather is fine, a little gentle exercise in the open air. Purgatives are not necessary, since as soon as the disease is over, the bowels regain their proper tone and become perfectly regular.—*Am. Practitioner.*

---

ALMOST A DEATH FROM CHLOROFORM.—Dr. Wm. Walling, of this city, reports to us an interesting case, in which death seemed imminent, from a drachm of chloroform taken into the stomach. The subject was a healthy young man, a student of medicine, who took the chloroform (by Dr. W.'s advice) for colic. In about twenty minutes after swallowing it, he complained of dizziness, and, a little while after, fell over, motionless and insensible. For a time, no respiratory movements were perceived, and the pulse at the wrist failed, and the carotid arteries beat languidly. For nearly an hour, the pulse was not more than 45, and occasionally below 40, and feeble. Hartshorn was held near his nose and applied to his face, with affusions of cold water. Suddenly he rushed up and spoke, and all the alarming symptoms passed off, greatly to the relief of his medical adviser. Physicians often prescribe chloroform internally, in drachm doses. This case shows that such doses may be followed by appearances frightful to the oldest practitioner.—*American Practitioner.*

---

ANTAGONISM OF MORPHIA AND ATROPIA.—A good example of the antagonism of these drugs is referred to in the *Medical Times and Gazette*, for Nov. 20, as occurring in the practice of M. Bêhier. In this case, an old man took a solution of sulphate atropia, prepared for ophthalmoscopic purposes, containing one-fifth of a grain. He experienced an acid taste in the throat, slight embarrassment in the movements of the tongue, muscular weakness, a difficulty in walking, soon amounting to impossibility, and disturbance of vision. M. Bêhier, knowing the antagonism of morphia and atropia (described by Gräfe in 1862), prescribed ten drops of laudanum every ten minutes. Each dose diminished the intensity of the symptoms. The patient took on the whole seventy-six drops — a dose which, if he had not previously taken the atropia, would have undoubtingly produced symptoms of poisoning by opium.—*Cincinnati Lancet and Observer.*

